# Knowledge domain and trends in treatment-resistant schizophrenia (TRS) research based on CiteSpace bibliometrics analysis

**DOI:** 10.3389/fphar.2024.1478625

**Published:** 2024-11-05

**Authors:** Haipeng Cai, Ruonan Du, Jianyi Zhang, Xin Wang, Wei Li, Kebing Yang, Zhiren Wang

**Affiliations:** Beijing Huilongguan Hospital, Huilongguan Clinical Medical School of Peking University, Beijing, China

**Keywords:** treatment-resistant schizophrenia, efficiency, clozapine, CiteSpace, bibliometrics analysis, global trends

## Abstract

**Background:**

Although the number of studies on treatment-resistant schizophrenia (TRS) has been increasing, the global research hotspots and future research trends have not yet been established.

**Objective:**

This study identify the hotspots of TRS research and predict future research trends using a bibliometric analysis.

**Methods:**

The Web of Science Core Collection was searched using the keyword “TRS”, econometric and co-occurrence analyses were conducted using CiteSpace and VOSviewer software, and the results were visualised. PRISMA reporting guidelines were used for this study.

**Results:**

In total, 912 publications were included in the analysis. The number of publications on TRS has shown an increasing trend over the past 20 years. The United States and University of London were the countries and institutions with the highest total number of publications, respectively. *Schizophrenia Research* was the journal with the highest number of articles. *American Journal of Psychiatry* was the most cited journal. Based on the results of this analysis, cognitive impairment, clozapine-resistant schizophrenia, early-onset schizophrenia, and early recognition of TRS will be hotspots for future research in this field.

**Conclusion:**

There has been an upward trend in the number of publications on TRS each year. However, issues such as how to use antipsychotics more efficiently to treat TRS and how to predict the emergence of TRS as early as possible are still in urgent need of research and are current challenges for clinicians. The results of this study not only predict and analyse future research hotspots but also help researchers identify appropriate research directions and partners.

## Introduction

Schizophrenia is a complex, severe, and progressive mental disorder characterised by a cluster of positive (such as hallucinations and delusions) and negative symptoms (such as diminished volition, emotional apathy, and social withdrawal) (Correll, 2020). This disorder not only affects the patient’s thinking, self-awareness, perception, emotion, language, and behaviour but may also increase all-cause mortality and the risk of suicide (Correll et al., 2022). Some researchers have proposed the concept of the schizophrenia spectrum, which refers to a group of mental disorders associated with schizophrenia that share similar symptoms and features. Thus, schizophrenia may be a continuum of symptoms and disorders, rather than an isolated disorder.

Evidence suggests that the proportion of individuals presenting with schizophrenia spectrum disorders before the age of 25 years is 47.8%, with peak onset at 20.5 years of age (Correll et al., 2017). Reports from the 2016 Global Burden of Disease (GBD) indicate that the prevalence of schizophrenia is increasing worldwide, growing to 21 million cases in 2016 (Charlson et al., 2018). A report from the GBD 2019 noted an increased prevalence of schizophrenia among 25–49-year-olds (Collaborators, 2020). Schizophrenia not only reduces the quality of life of patients, but also creates a huge burden on families and society (Kennedy et al., 2014).

Treatment-resistant schizophrenia (TRS), a clinical problem with heterogeneous presentations, is considered the most severe subtype of schizophrenia. The concept of TRS first appeared in 1966 (Itil et al., 1966), its definition remains vague, and the current definitions of TRS are broadly consistent across different treatment guidelines (Lehman et al., 2004; Hasan et al., 2012; Howes et al., 2017). TRS is generally defined as the persistence of positive symptoms (e.g., delusions, hallucinations, and disturbances in speech and behaviour) in patients who have been administered ≥2 different non-clozapine antipsychotics with an adequate dosage, duration of medication, and adherence. Approximately 30% of patients with a diagnosis of schizophrenia are affected by TRS, and they show partial/no response to initial antipsychotic medications (Meltzer, 1997). A further 10%–60% of patients show an initial response to drug treatment, but develop resistance to drugs as the condition recurs or over time (Kane, 2013). Additional data support the above notion that, with relapse of psychotic symptoms and successive changes to different antipsychotic medications, only approximately 9% of patients respond to a third non-clozapine antipsychotic medication (Kinon et al., 1993).

Currently, pharmacotherapy is the mainstay of treatment for TRS, and there are several commonly used modalities, such as increasing the duration of antipsychotic medication, increasing the dosage of non-clozapine antipsychotics, and combining multiple medications (addition of an affective stabiliser and two or more non-clozapine antipsychotic medications). However, these regimens are limited in their efficacy and may delay the use of clozapine, or even affect the antipsychotic clinical response in patients who do not respond to medication (Taylor et al., 2003; Wheeler, 2008; Howes et al., 2012; Alessi-Severini et al., 2013). Clozapine is the only drug currently approved by the Food and Drug Administration (FDA) for TRS (Kane et al., 2019). However, owing to its potentially life-threatening side effects (e.g., agranulocytosis), the use of clozapine has been limited in certain countries. In addition to medication, non-pharmacologic treatments such as electroconvulsive therapy (ECT) have also been shown to be effective for TRS. However, the aforementioned pharmacologic and non-pharmacologic approaches are not effective for all patients with TRS, which provides additional evidence of the heterogeneity in TRS and suggests that the underlying neurobiological mechanisms of TRS may be different from those of schizophrenia, for which medication is effective. However, to date, these issues have not been conclusively addressed.

An increasing number of studies have begun to focus on the possible neurobiological mechanisms of TRS, effective therapeutic regimens, and the exploration of novel drugs (e.g., glutamate modulators); however, global research hotspots and future research trends in this area are not yet established. Bibliometrics analyses and interprets literature data using mathematical and statistical methods to reveal the structure, trends, patterns, and impact of a particular field of study and visualises the results (Hicks et al., 2015). It helps discover new areas of knowledge and research directions by identifying key concepts and themes in the literature (Ninkov et al., 2022). The aim of this study is to identify the global trends in research on TRS through bibliometric and visualization analyses, to explore the current research state, and to identify the research hotspots and development trends in this field in recent 20 years.

## Materials and methods

### Data sources

**FIGURE 1:**
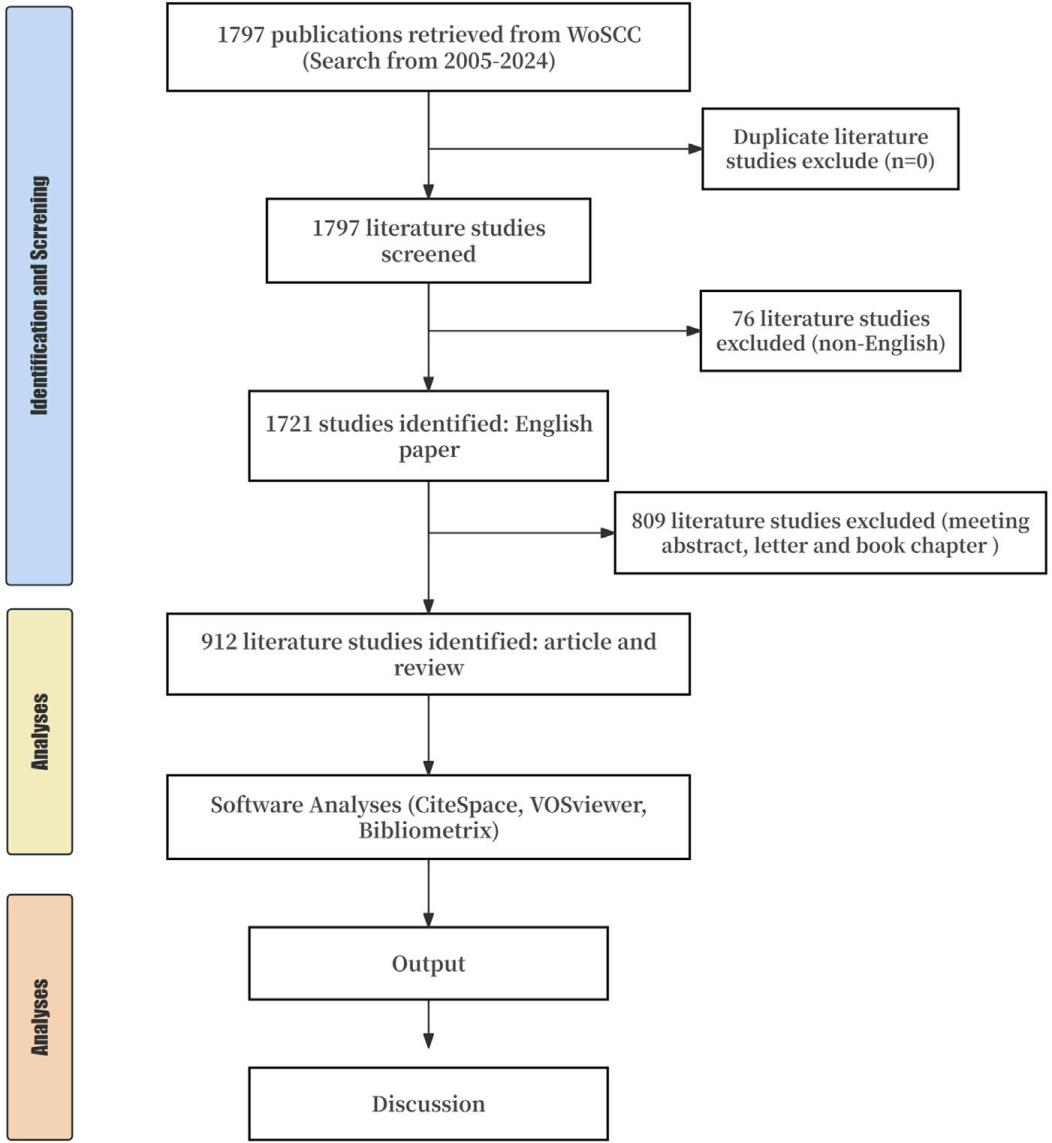
Flow chart of the study.

The Web of Science Core Collection (WoSCC) database is one of the most commonly used comprehensive databases for bibliometric analysis and it contains a multidisciplinary index of scholarly literature abstracts that include multiple datasets. We searched the WoSCC database on 24 June 2024. PRISMA reporting guidelines were used for this study (Figure 1). We set the literature search formula to “(title [TI]=(“treatment-resistant” OR “treatment resistant” OR “treatment-refractory” OR “treatment-refractory” OR “Antipsychotic-resistant” OR “Antipsychotic-refractory” OR refractory OR TRS) AND TI=(schizophrenia)) OR (author keyword [AK]=(“treatment-resistant” OR “treatment resistant” OR “treatment-refractory” OR “treatment-refractory” OR “Antipsychotic-resistant” OR “Antipsychotic-refractory” OR refractory OR TRS) AND AK=(schizophrenia))”. We set the types of literature as articles and reviews, excluding other types (such as conference, correspondence, and books). We set the language type to English.

A total of 1,797 TRS documents were retrieved using the search formula above. After setting the language to English, a total of 1,721 studies were found. After the exclusion of other studies, 912 original studies and reviews were included in the final analysis.

### Introduction of bibliometric instruments

CiteSpace (https://citespace.podia.com/) is a scientific literature analysis tool developed by Prof. Chaomei Chen of Drexel University. It is based on the Java programming language and is free of charge to users. The design of CiteSpace is based on a number of theories, including the idea of co-occurrence clustering, structural hole theory, optimal information theory, and the theory of structural variation, which focus on analysing the research frontiers of disciplines as well as their evolution trends and internal connections. CiteSpace uses co-occurrence clustering to analyse information in scientific literature, such as keywords, authors, and institutions, to reveal the implicit patterns and laws of the knowledge structure of the discipline through the co-occurrence relationship of these information units and to detect the influence of the literature.

VOSviewer (https://www.vosviewer.com/) is a scientific knowledge mapping software that is one of the most important tools for bibliometric analysis and visualisation of scientific knowledge and it is mainly used to construct and visualise bibliometric networks. This software was developed in 2009 by van Eck and Waltman at the Center for Science and Technology Research at Leiden University, Netherlands. The core concept of software design is co-occurrence clustering, which reveals the relationships between different research entities (such as journals, literature citations, and keywords) through co-occurrence relationships and provides multiple visualisation modes.

### Data analysis

In this study, we used the WoSCC database to retrieve relevant literature using the above search formula. We preliminarily obtained data on annual publication volume, impact factor (IF), and h-index of publications on WoSCC, and used Microsoft Excel (Microsoft Corp., Redmond, WA, United States) to organise and visualise the data. The search results were filtered by language and the type of literature. Relevant literature was saved in plain text format and imported into CiteSpace v6.2 R6. Duplicate studies were eliminated using a data-cleaning function.

Nodes were selected according to the type of analysis to be performed with the following parameter settings: time slice: January 2005–June 2024; term source: title, abstract, author keyword, and keyword; node type: author, institution, country, keyword, citation, citing author, and citing journal; and selection criterion: Top N = 50. Click on the “GO” button to start the analysis. Relevant results were generated using CiteSpace, and visual images were generated simultaneously. The larger the node in the obtained image, the higher the frequency of occurrence of the research entity (such as researcher and keyword) represented by that node.

In addition, we used VOSviewer software to analyse the national knowledge networks and keyword co-occurrence networks of related studies and visualised the results using the R language package, Bibliometrix (https://www.bibliometrix.org/home/).

## Results

### Distribution of annual publication

**FIGURE 2:**
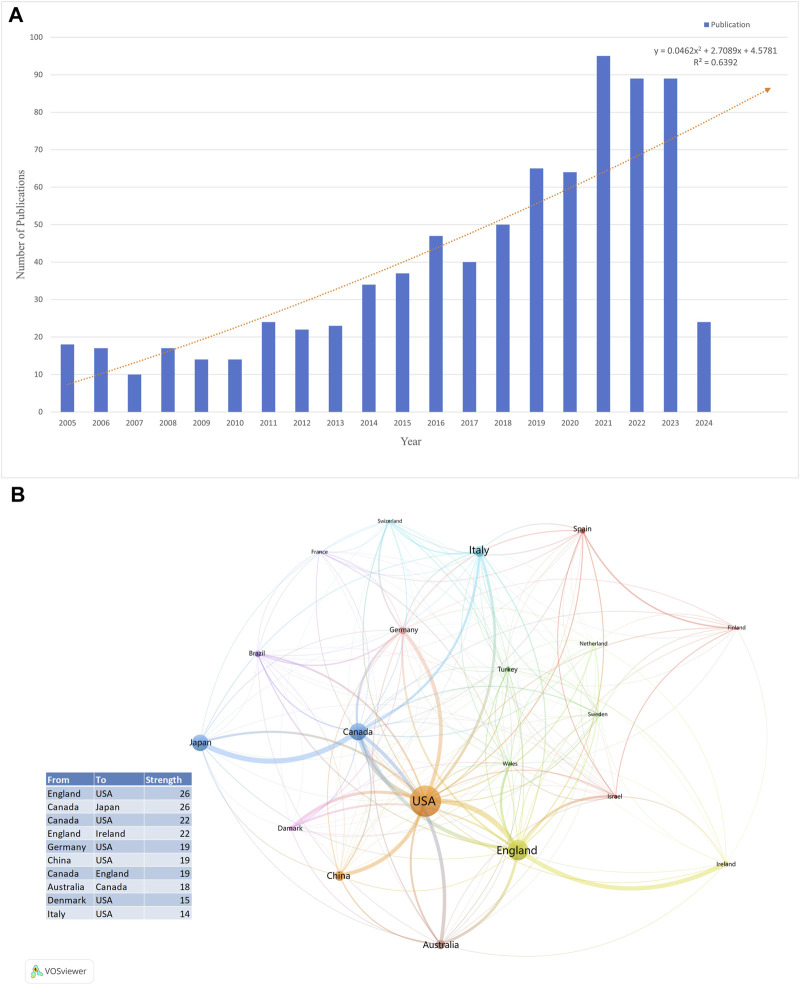
**(A)** The number of publications from 2005 to 2024. **(B)** Overlay visualization of country/region for TRS research.

Changes in the number of papers are important indicators of trends in this field. From January 2005 to June 2024, a total of 804 articles and reviews with the subject term “TRS” were published, and the annual number of publications showed an increasing trend, indicating that an increasing number of researchers are focusing on TRS. We visualised the annual number of publications using Microsoft Excel 365 (Microsoft Corp.) (Figure 2A) and added a trend line to forecast the number of articles posted. The predicted growth model equation was y = 0.0462x^2^ + 2.7089x + 4.5781, R^2^ = 0.6392, with x representing (forecast year - 2005) and y representing the predicted number of publications per year.

### Analysis of scientific collaboration network

**FIGURE 3:**
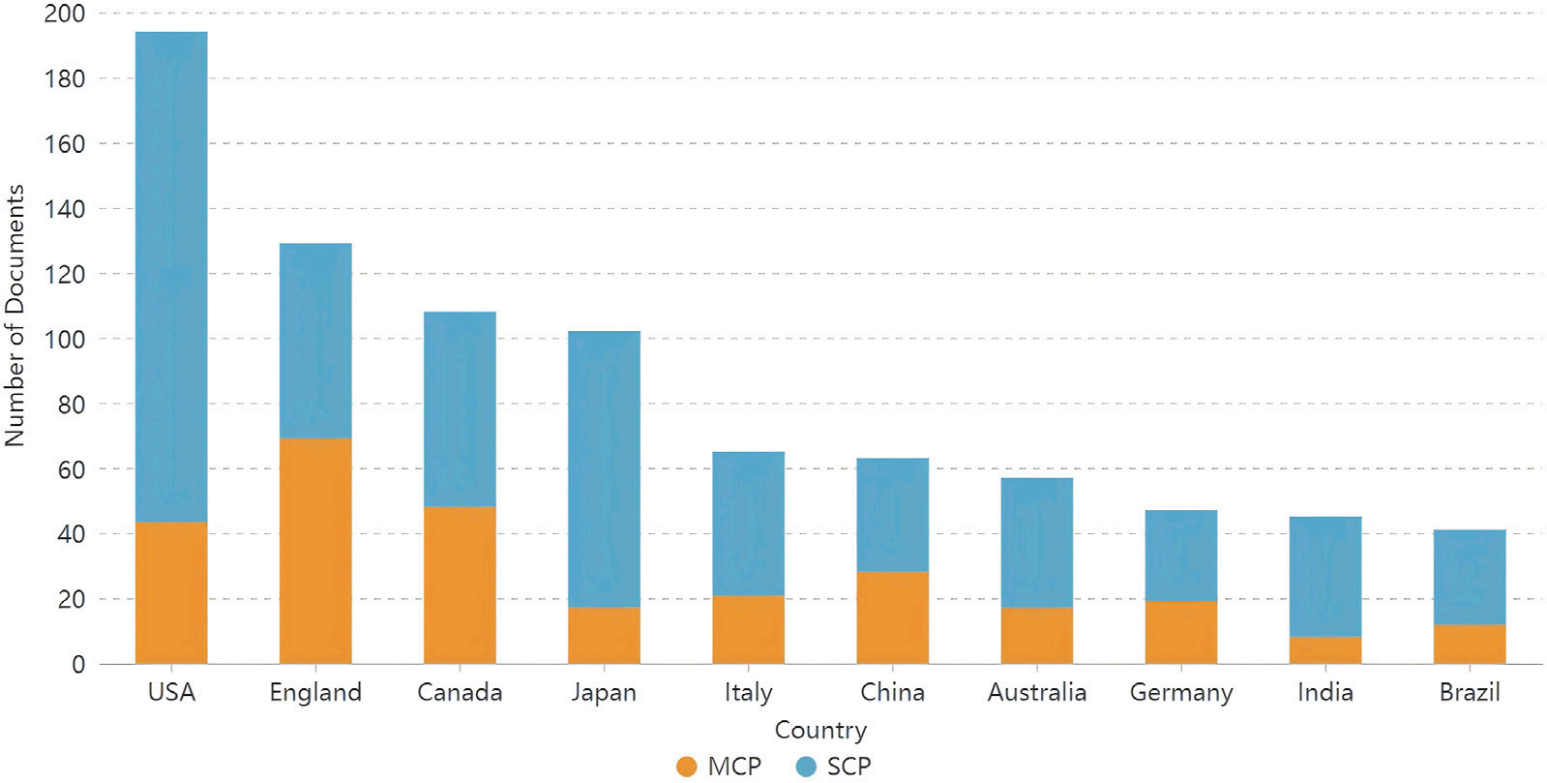
Corresponding author’s countries of documents related to TRS.

A total of 67 countries/regions published articles on TRS. Table 1 lists the top 10 countries in terms of the number of publications, with the United States having the highest number of publications in this field (n = 194; 21.27%), followed by England (n = 129; 14.14%) and Canada (n = 108; 11.84%). The top 10 country/region collaborations are listed in Figure 2B, with collaborations between England, the United States, Canada, and Japan being the most common. In addition, we analysed the compositions of the corresponding authors of the articles. Figure 3 shows the composition of corresponding authors in articles published in different countries; SCP stands for single-country publications and MCP stands for multiple-country publications, which reflects the intensity of foreign collaboration in each country in another way. Figure 3 shows that England, Canada, and China have stronger collaborations with foreign researchers.

**TABLE 1:** The top 10 productive countries/regions of the topic.

Country	Number of publications	Percentage (%)
United States	194	24.12
England	129	16.04
Canada	108	13.43
Japan	102	12.69
China	66	8.21
Italy	64	7.96
Australia	57	7.09
Germany	47	5.85
India	45	5.60
Brazil	41	5.10

The top 10 institutions by number of publications are listed in Table 2, with the University of London, King’s College London, and the Centre for Addiction & Mental Health - Canada, being the top three institutions in terms of publications in this area. The centrality value for each institutional node was calculated using the CiteSpace software, which was be used to measure the node’s influence and connectivity in the network; a larger centrality value for an institution means that the institution has more extensive collaboration with other institutions. We found that the top three institutions in terms of centrality were the Centre for Addiction and Mental Health - Canada, King’s College London, and the University of Toronto.

**TABLE 2:** Top 10 institutions according to publications and the corresponding centrality.

Rank	Name	Number	Centrality
1	University of London	90	0.13
2	King’s College London	88	0.14
3	Centre for Addiction and Mental Health - Canada	48	0.17
4	University of Toronto	46	0.11
5	South London and Maudsley NHS Trust	38	0.02
6	Keio University	36	0.07
7	Northwell Health	29	0.21
8	Chiba University	26	0.04
9	University of Naples Federico II	23	0.02
10	Aarhus University	15	0.01

Based on the number of publications, we listed the names of the top 10 authors, their institutions, and their h-indices (Table 3). James H. Maccabe was the author with the highest number of publications. Four of the top 10 authors with the most publications were from the University of Naples Federico II, indicating that this institution has a significant influence in the field of TRS. The h-index is a measure of the academic influence of scholars, which is based on the number of citations and papers of a scholar, and it reflects the academic influence of scholars in a relatively objective way. Table 3 shows that the authors with the highest h-index among the top 10 authors in terms of publications were Remington and Gray from Canada.

**TABLE 3:** Top 10 authors according to publications, the corresponding institution and h-index.

Rank	Author	Publications	Institution	h-index
1	Maccabe, James H.	28	King’s College London	44
2	de bartolomeis, Andrea	26	University of Naples Federico II	2
3	Iasevoli, Felice	19	University of Naples Federico II	37
4	Iyo, Masaomi	18	Chiba University	61
5	Remington, Gary	17	Centre for Addiction and Mental Health - Canada	67
6	Barone, Annarita	17	University of Naples Federico II	9
7	Kanahara, Nobuhisa	15	Chiba University	23
8	Lally, John	13	King’s College London	25
9	Graff-guerrero, Ariel	12	University of Toronto	39
10	Vellucci, Licia	12	University of Naples Federico II	8

### Analysis of journals, co-cited journals, and co-cited references

According to the WoSCC database, articles on TRS were published in 671 journals. The top 10 journals and co-cited journals were mainly in the fields of psychiatry, pharmacology, and brain science (Table 4), and most journals were in the Quartile 1 (Q1) region of the Journal Citation Reports (JCR) partition. The JCR is an annual journal evaluation report published by Clarivate Analytics that provides journal IFs and various other evaluation metrics to help researchers and academic institutions assess the relative importance and impact of journals within their subject areas. The JCR typically categorises journals within each subject area into four quartiles (Q1, Q2, Q3, and Q4), with each zone containing approximately 25% of the journals in that area. Journals in Q1 had the highest IF, whereas journals in Q4 had a relatively low IF. When choosing journals in which to publish research results, researchers can refer to the JCR partitions to select high-impact journals in their respective fields.

**TABLE 4:** Top 10 journals and co-cited journals.

Items	Ranking	Name	Counts	IF2023	JCR
Co-cited Journals	1	American journal of psychiatry	651	17.7	Q1
	2	Schizophrenia research	628	4.5	Q2
	3	Schizophrenia bulletin	589	6.6	Q1
	4	Journal of clinical psychiatry	501	5.3	Q2
	5	Archives of general psychiatry	470	NA	NA
	6	The british journal of psychiatry	443	10.5	Q1
	7	Biological psychiatry	438	10.6	Q1
	8	Psychiatry research	397	11.3	Q1
	9	Acta psychiatrica scandinavica	377	6.7	Q1
	10	Progress in neuro-psychopharmacology and biological psychiatry	323	5.6	Q1
Journal	1	Schizophrenia research	61	4.5	Q2
	2	Journal of clinical psychopharmacology	38	2.9	Q3
	3	Journal of psychopharmacology	36	4.1	Q2
	4	Psychiatry research	35	11.3	Q1
	5	Journal of clinical psychiatry	34	5.3	Q2
	6	Frontiers in psychiatry	26	4.7	Q2
	7	Asian journal of psychiatry	18	9.5	Q1
	8	Journal of psychiatric research	18	4.8	Q2
	9	BMC psychiatry	17	4.4	Q2
	10	International clinical psychopharmacology	16	2.6	Q3

The journal with the highest number of publications was *Schizophrenia Research*, followed by *Journal of Clinical Psychopharmacology* and *Journal of Psychopharmacology*. An analysis of journal co-citations revealed the contribution of each journal to the field. The top three journals based on co-citation counts were *American Journal of Psychiatry* (651), *Schizophrenia Research* (628), and *Schizophrenia Bulletin* (589). Table 5 shows information on the top 10 highly cited articles.

**TABLE 5:** Top 10 highly cited references.

Rank	Author	Title (publication year)	Journal (IF2023)
1	Howes O. D. et al.	Treatment-resistant schizophrenia: treatment response and resistance in psychosis (TRRIP) working group consensus guidelines on diagnosis and terminology (2017)	American journal of psychiatry (17.7/Q1)
2	Siskind D. et al.	Clozapine response rates among people with treatment-resistant schizophrenia: data from a systematic review and meta-analysis (2017)	Canadian journal of psychiatry (5.32/Q2)
3	Lally J. et al.	Two distinct patterns of treatment resistance: clinical predictors of treatment resistance in first-episode schizophrenia spectrum psychoses (2016)	Psychological medicine (6.985/Q1)
4	Kane J. M. et al.	Clinical guidance on the identification and management of treatment-resistant schizophrenia (2019)	Journal of clinical psychiatry (5.3/Q2)
5	Nucifora F. C. et al.	Treatment resistant schizophrenia: clinical, biological, and therapeutic perspectives (2019)	Neurobiology of disease (6.1/Q1)
6	Potkin S. G. et al.	The neurobiology of treatment-resistant schizophrenia: paths to antipsychotic resistance and a roadmap for future research (2020)	NPJ Schizophrenia (5.46/Q2)
7	Egerton A. et al.	Dopamine and glutamate in antipsychotic-responsive compared with antipsychotic-nonresponsive psychosis: a multicenter positron emission tomography and magnetic resonance spectroscopy study (STRATA) (2021)	Schizophrenia bulletin (6.6/Q1)
8	Siskind D. et al.	Clozapine v. first- and second-generation antipsychotics in treatment-refractory schizophrenia: systematic review and meta-analysis. (2016)	The british journal of psychiatry (10.5/Q1)
9	Kennedy J. L. et al.	The social and economic burden of treatment-resistant schizophrenia: a systematic literature review (2014)	International clinical psychopharmacology (2.6/Q3)
10	Bachmann C. J. et al.	International trends in clozapine use: a study in 17 countries (2017)	Acta psychiatrica scandinavica (6.7/Q1)

### Changes in trends of research disciplines

**FIGURE 4:**
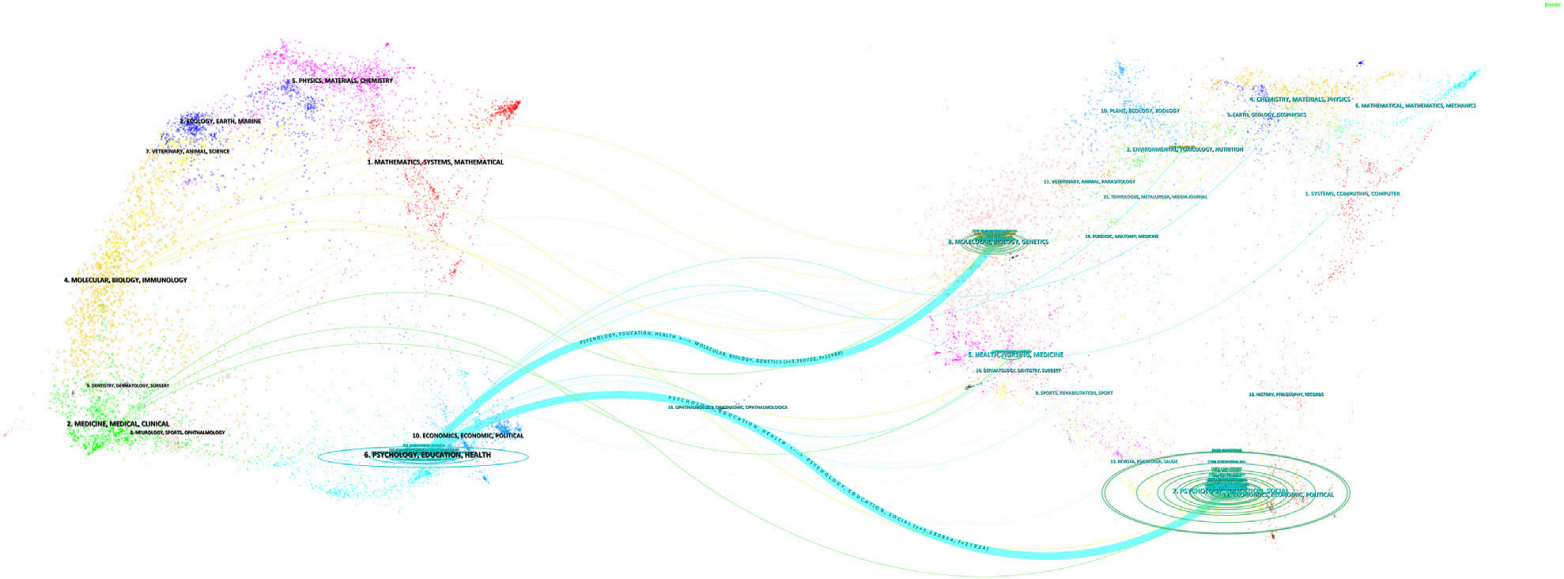
Dual-map overlay of article citations for TRS research.

CiteSpace’s dual-map overlay analysis feature can overlay two scientific knowledge graphs to compare and analyse changes in the network structure under different topics or time periods. This feature was used to visualise disciplinary intersections in the TRS field. Figure 4 shows the citation relationships between journals, with citing journals on the left side of the image, cited journals on the right side of the image, and the thicker two blue lines representing the citation paths for the core of the field. The citation path indicated that articles in journals with the theme PSYCHOLOGY/EDUCATION/HEALTH were mostly cited by articles in journals with the related theme MOLECULAR/BIOLOGY/EDUCATION/SOCIAL/GENETIC/PSYCHOLOGY.

### Analysis of co-occurring keywords

**FIGURE 5:**
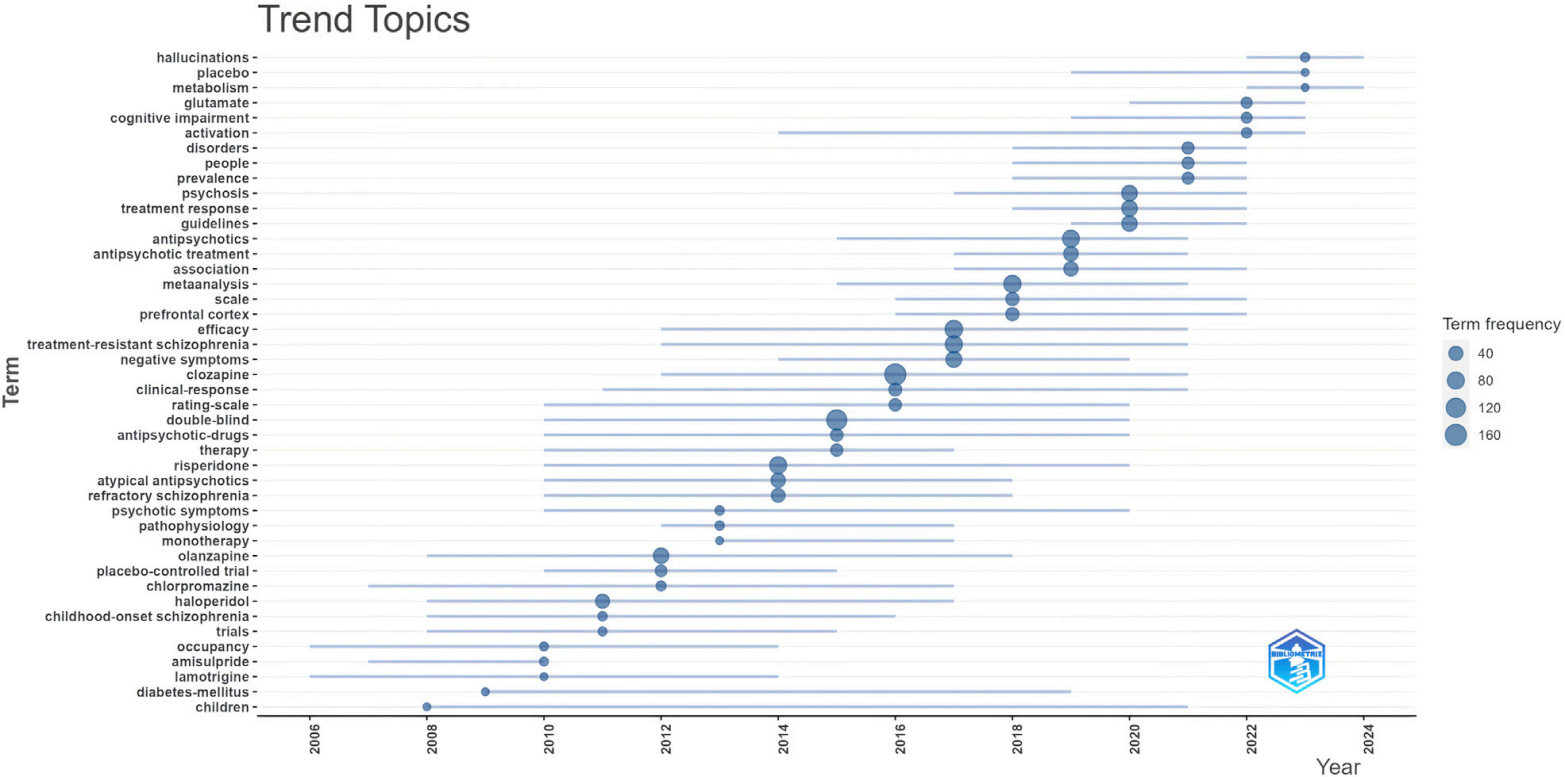
Trends topic based on keywords frequency of occurrence more than 40.

**FIGURE 6:**
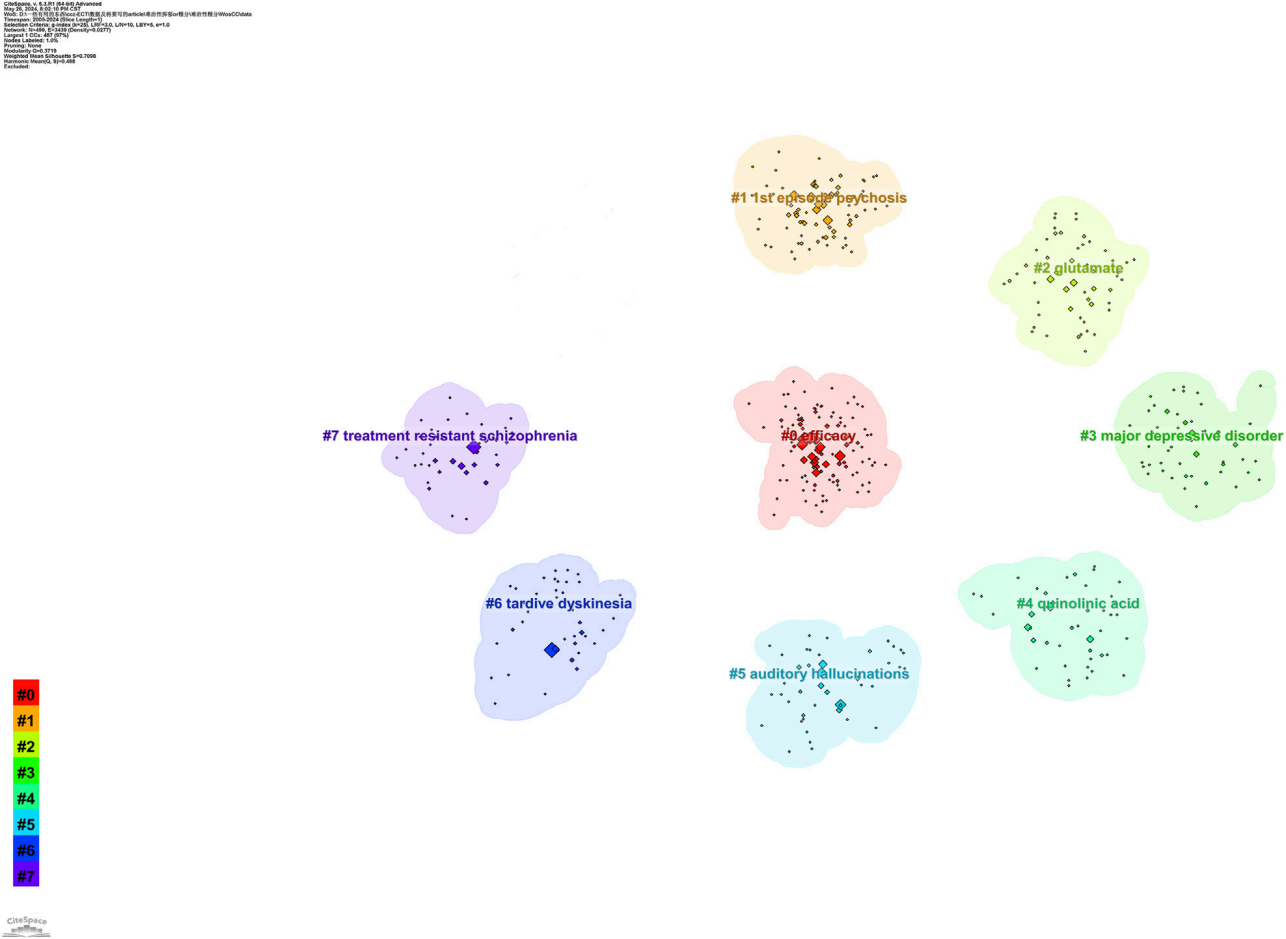
Clusters of co-occurring keywords in TRS research.

We listed the top ten keywords based on their frequencies of occurrence (Table 6). The most frequently occurring keyword was “treatment-resistant schizophrenia,” followed by “clozapine,” “cognitive impairment,” and “efficacy.” We visualised keyword trends using the “Biblometrix” R package and Figure 5 shows keywords with more than 40 occurrences. The size of the circle indicates the frequency of occurrence; the larger the circle, the more frequent the occurrence. The most frequent keywords in the last 5 years were “treatment response,” “psychosis,” and “prevalence.” We clustered the co-occurring keywords to find hot research topics, and the results are shown in Figure 6. Based on an analysis of co-occurring keywords and clustering results, efficacy, antipsychotics, and metabolism were popular research topics.

**TABLE 6:** Top 10 keywords in terms of frequency in the TRS research.

Rank	Keyword	Frequency
1	treatment-resistant schizophrenia	387
2	clozapine	160
3	cognition impairment	136
4	efficacy	86
5	risperidone	78
6	antipsychotics	77
7	negative symptoms	76
8	metabolism	62
9	psychosis	56
10	guidelines	55

### Analysis of burst citation

**FIGURE 7:**
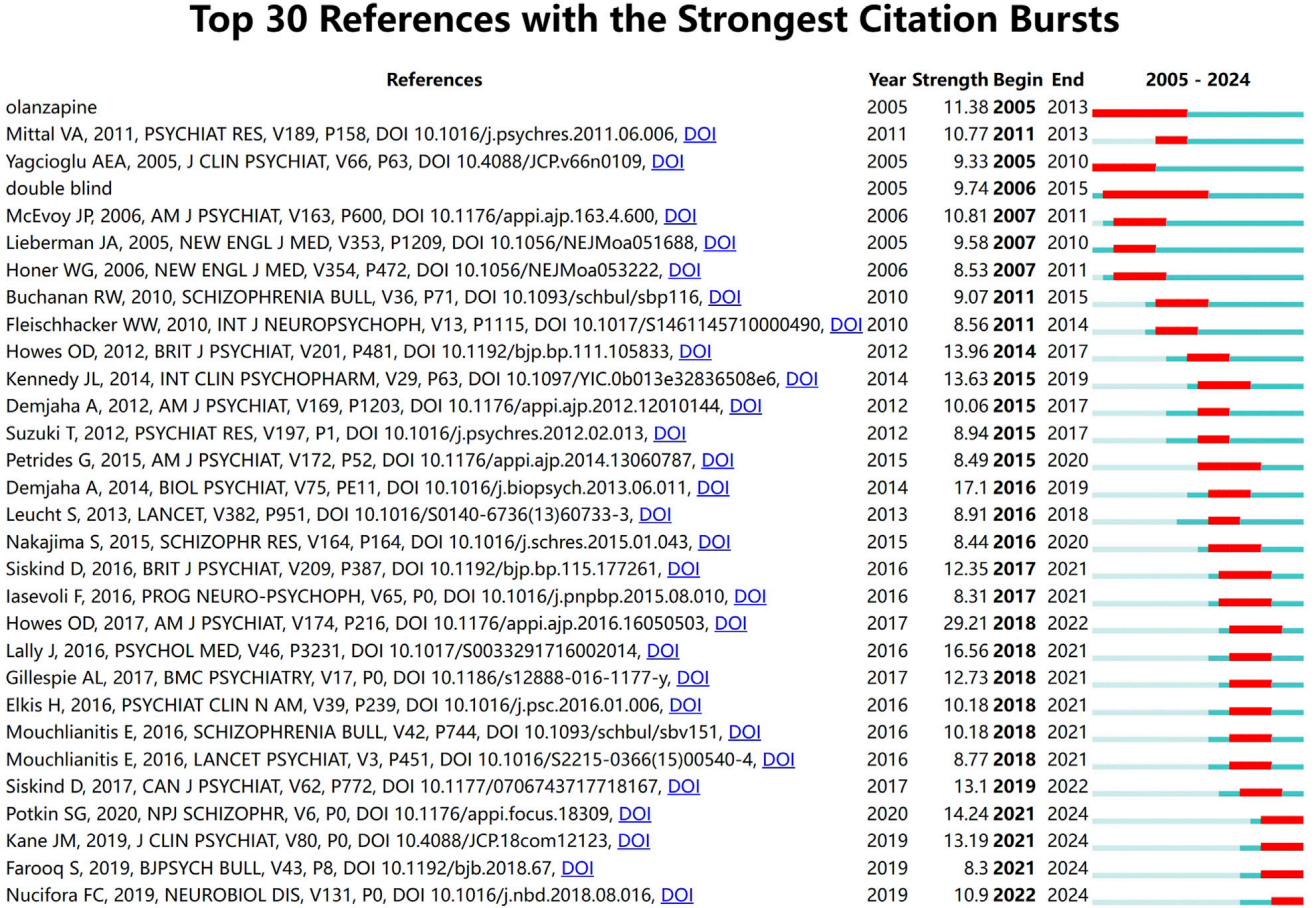
Top 30 references with the strongest citation bursts.

Reference citation bursts illustrate the evolution of the knowledge domain. The blue line represents the period from 2005 to 2024, and the red line represents the duration of the keyword burst. Figure 7 lists the 30 references with high citation volumes. The duration of a citation outburst is indicated by a red line. There were four studies in which the outbreak lasted until 2024 (Farooq et al., 2019; Kane et al., 2019; Nucifora Jr et al., 2019; Potkin et al., 2020). The strongest outbreak intensity (Howes et al., 2017) was reported in a 2016 article published in the *American Journal of Psychiatry* by Howes et al. Their research team defined resistance by reviewing multiple studies on antipsychotic medications for the treatment of TRS and proposed consistent guidelines that provide a basis for research and clinical translation.

## Discussion

This study is the first to use bibliometrics to systematically sort and analyse academic literature in the field of TRS, revealing the research evolution and global trends in the field since 2005. Through a bibliometric lens, this study provides new insights and directions for academic research in the TRS field. The results of the study not only help academics better understand the current state of research in the field of TRS but also provide important guidance and reference for future research. By reducing the blindness of research topic selection, the findings of this study are expected to contribute to the accumulation of knowledge and academic development in the field of TRS and provide strong support for researchers in this field.

### Global trends in TRS

Our analysis showed that the number of publications in this field has increased since 2005. The United States had the highest number of publications among a large number of countries/regions, and its collaboration with other countries was more prominent, with larger contributions in this field. The University of London had the most publications in this area of all institutions, followed by King’s College London and the Centre for Addiction and Mental Health in Canada. The author with the most publications was James H. Maccabe from King’s College London. The author with the highest h-index among the top 10 postings was Remington Gray, from the Centre for Addiction & Mental Health - Canada. Although partial collaboration exists between various countries and institutions, the intensity of this collaboration needs to be strengthened. Our study helps researchers understand the research directions of different countries and institutions and find suitable partners.

### Status of publications

Most articles in this field were published in journals related to psychiatry, pharmacology, and brain sciences. Among them, the highest number of articles were published in *Schizophrenia Research*, the official journal of the International Society for the Study of Schizophrenia, which brings together biological, clinical, and psychological research that contributes to the understanding of the biology and treatment of schizophrenia via novel papers to facilitate the synthesis of findings from all disciplines involved in improving the prognosis of people with schizophrenia. The majority of the 10 most co-cited articles dealt with disease management in TRS and mechanisms of antipsychotic resistance, with six of these published in Q1 journals.

The most co-cited article was a consensus guideline on the diagnosis and terminology of TRS developed by the Treatment Response and Resistance in the Psychosis Working Group. This research extracted a definition of treatment resistance through a systematic review of randomised antipsychotic clinical trials between 1980 and 2016 and identified key criteria through multiple methods, culminating in a conference to reach consensus. Nearly half of the included studies did not use fully operationalised criteria; therefore, these studies cannot be fully replicated. Most of these studies used different criteria, which made between-study comparisons difficult. In the current context of inconsistencies in the definition and management of TRS, the group addressed the differences in the definition of TRS and treatment response by establishing a set of consensus criteria to facilitate future research and improve clinical practice, providing a benchmark for future clinical work and research.

### Hotpots and frontiers

High-frequency keywords and co-occurring words usually reflect research hotspots or major concerns in the field. Research trends and topic evolution can be identified by analysing the frequency of keyword appearances. By analysing keyword clusters, cluster analysis can help identify emerging research frontiers and predict possible directions for research development. We analysed the results of co-occurring words and cluster analysis to predict future research hotspots in the field, as follows:

#### TRS and cognitive impairment

Cognitive impairment is one of the core features of people with schizophrenia (Reichenberg et al., 2009; McCleery and Nuechterlein, 2019), including neurocognitive domains (e.g., attention, processing speed, verbal and visual memory, working memory, and executive functioning (Fervaha et al., 2014; Käkelä et al., 2014; Quee et al., 2014)), as well as social cognitive domains (e.g., emotional control and social perception (Minzenberg et al., 2004; Hill et al., 2010)), and is associated with functional deficiencies (Green, 2016). This directly or indirectly reduces the quality of life of patients (Buchanan et al., 1994; Gallhofer et al., 1996; Fagerlund et al., 2004) and is one of the main limiting factors in the rehabilitation of mental disorders (Green et al., 1997; Green et al., 2002; Goldberg et al., 2007). Some studies have found that cognitive impairment may be present early in the disease, even before the onset of psychiatric symptoms (Bora et al., 2017; Glenthøj et al., 2020). As time progresses, most cognitive functions may improve longitudinally or remain unchanged over the disease trajectory, while a minority of cognitive functions progressively deteriorate (Bozikas and Andreou, 2011; Chan et al., 2023). A recent meta-analysis found that patients with TRS had significant deficits in all domains of cognitive functioning compared with patients treated effectively with antipsychotics (Millgate et al., 2022). However, results of current studies on the effects of antipsychotics on cognitive function in patients with schizophrenia are inconsistent. A recent network meta-analysis by Baldez et al. analysed randomised controlled trials using different antipsychotic medications to compare their effects on cognitive function in patients with mental disorders. They found that different antipsychotics improved cognitive function; however, the cognitive domains were improved by different drugs. For example, olanzapine improved several cognitive functions, while clozapine performed poorly in executive functioning and verbal learning (Baldez et al., 2021). Another meta-analysis comparing the cognitive effects of second-generation antipsychotics in patients with schizophrenia found that clozapine negatively affected verbal working memory and executive function but improved verbal fluency (Nielsen et al., 2015). However, these studies did not limit the population to patients with TRS and did not consider the level of cognitive function in patients before and after medication. As the only effective antipsychotic drug for TRS, is important to clarify the effect of clozapine on cognitive function in patients with TRS in future studies, both in terms of medication selection and the prognosis of TRS.

In addition to pharmacological interventions, studies on psychosocial interventions for cognitive deficits in patients with schizophrenia have attracted the attention of most researchers, with cognitive remediation therapy (CRT) showing the most evidence of effectiveness; however, few studies have been conducted on the application of CRT to TRS. CRT is a behavioural training-based intervention designed to improve cognitive processes (Bowie et al., 2020). Martini et al. evaluated the efficacy of CRT in TRS for the first time and compared the cognitive performance of patients with TRS and patients without TRS before and after CRT. Their study found that patients with TRS showed more pronounced improvements in verbal memory and executive functioning than patients with schizophrenia without drug resistance.

In addition, some non-pharmacological treatments based on the promotion of neuroplasticity, such as physical exercise (especially aerobic exercise), have been shown to be beneficial in improving social and cognitive functioning in patients with schizophrenia (Rosenbaum et al., 2014; Dauwan et al., 2016; Firth et al., 2017). A randomised controlled study by Shimada et al. in Japan confirmed that a treatment regimen of aerobic exercise superimposed on conventional treatment (medication and other rehabilitation programs) is more effective and safe than conventional treatment in improving cognition, internal motivation, and interpersonal relationships in patients with schizophrenia (Shimada et al., 2019). However, there is no evidence of aerobic exercise in patients with TRS.

Cognitive impairment has a significant impact on symptom recovery and social functioning rehabilitation in patients with TRS; thus, improvement of cognitive impairment in patients with TRS remains a pressing issue. Despite this, existing evidence for methods of cognitive improvement in TRS is limited, further highlighting the urgent need for new and effective interventions and further exploration of the mechanisms of cognitive deficits in patients with TRS, as well as the optimisation of current conventional treatment protocols. Therefore, the improvement of cognitive deficits in patients with TRS will be an important research topic for future studies.

#### Clozapine-resistant schizophrenia

Medication is one of the preferred treatments for schizophrenia, but approximately one-third of people with schizophrenia respond poorly to antipsychotic medications (first- or second-generation antipsychotics) (Kane et al., 2019; Wagner et al., 2019), with a <20% reduction in positive and negative syndrome scale scores before and after retreatment. The poor efficacy of antipsychotic medications in patients with TRS is usually associated with poorer prognosis, higher risk of suicide, and poorer social functioning and quality of life, placing a significant economic burden on the patient’s family and society (Kane et al., 2019).

Clozapine is the first second-generation antipsychotic, the first to treat TRS effectively, and the only FDA-approved drug for TRS treatment (Pandey and Kalita, 2022). Unlike traditional first-generation antipsychotics, clozapine has a short duration of action on the D2 receptors, rapid dissociation, and a mechanism of action involving multiple receptors (5-HT2a, alpha-adrenergic, and NMDA receptors). In a real-world study by Tiihonen et al., 29,823 patients with schizophrenia were followed up for rehospitalisation and treatment outcomes, and the results showed that clozapine and long-acting injectable antipsychotics were the most advantageous medications for preventing relapses in schizophrenia (Tiihonen et al., 2017). The results of a recent study showed that patients with TRS who started clozapine later (≥20 years from diagnosis of schizophrenia to initiation of clozapine) had a significantly higher risk of rehospitalisation than those who started treatment earlier (Hatano et al., 2023). In other words, the delayed use of clozapine may increase the risk of long-term hospitalisation in patients with schizophrenia. However, this was a retrospective cohort study based on medical records, which had incomplete data, selection bias, and a small sample size. Therefore, the findings cannot be generalised to the entire population of patients with TRS. Although most studies now consider clozapine as an effective therapeutic agent for TRS, some have reached different conclusions. A network meta-analysis by Mishra et al. that summarised 47 randomised controlled studies showed that combining escitalopram, glycine, or mianserin with antipsychotics was superior to clozapine treatment in patients with TRS. This seems inconsistent with the general consensus in clinical practice (Mishra et al., 2024). It has been shown that about 40%–70% of patients with TRS are poorly treated with single-agent clozapine (Muscatello et al., 2014; Siskind et al., 2017; Roerig, 2019), and the concept of clozapine-resistant schizophrenia (CRS) has been proposed by some researchers. The current key elements regarding CRS include maintenance of a therapeutic dose of 200–500 mg/d, blood levels ≥350 ng/mL, duration of treatment of at least 2–3 months, and good adherence to treatment (ability to take more than 80% of the prescribed dose), but moderately-severe disease and functional impairment persisting despite adequate treatment with clozapine (Mouaffak et al., 2006; Tranulis et al., 2006; Lee et al., 2015). Current research on CRS has provided less high-quality evidence to support the idea that other psychotropic medications and non-pharmacologic treatments can increase the efficacy of clozapine. The addition of another antipsychotic medication to clozapine is currently one of the most common clinical treatments for CRS (Buckley et al., 2001; Pai et al., 2012; Muscatello et al., 2014; Lally and Gaughran, 2019). However, several studies have shown that clozapine in combination with another antipsychotic does not significantly improve overall symptom severity or positive symptom severity (Miyamoto et al., 2015; Faden and Citrome, 2019; Lally and Gaughran, 2019; Roerig, 2019). A few studies on the effects of clozapine in combination with other antipsychotics on negative and depressive symptoms have been published, and no consistent conclusions have been reached. There is less reliable evidence that antidepressants and mood stabilisers increase the efficacy of clozapine. In addition to studies on psychiatric medications that increase the efficacy of clozapine, studies on non-pharmacological treatments, such as ECT, to assist in the treatment of TRS, have begun to receive attention from researchers. Results show that ECT is an effective augmentation strategy (especially when medications do not improve positive symptoms) (Lally and Gaughran, 2019; Roerig, 2019; Wagner et al., 2019), and the modality is fast-acting and relatively safe in combination with antipsychotics. However, ECT has been poorly studied in patients with CRS, and further confirmation of its efficacy in large-sample, rigorously-designed trials is warranted. Moreover, repetitive transcranial magnetic stimulation therapy does not seem to be effective in the treatment of CRS (Wagner et al., 2019; Wagner et al., 2021). A randomised controlled study by Morrison et al. evaluated the efficacy of cognitive therapy (CBT) in patients with CRS and followed them for 21 months. The results showed that CBT provided a statistically significant improvement in overall symptoms in patients with CRS at the end of treatment compared with conventional treatment, but the follow-up results showed that CBT did not produce a lasting improvement in overall symptoms in patients with CRS (Morrison et al., 2018). The results of a recent meta-analysis suggest that CBT is effective in treating positive symptoms in clozapine-resistant patients; however, its efficacy in treating negative symptoms is uncertain (Polese et al., 2019).

CRS may be the most challenging type of schizophrenia to treat, but there is insufficient evidence to provide uniform and effective recommendations for treatment regimens for clozapine-resistant patients. Therefore, optimising clozapine treatment and exploring augmentation therapies for clozapine will be a hot topic for future research. In addition, negative and cognitive symptoms in patients with TRS appear to be less responsive to medication and have a more protracted course than those with positive symptoms. Therefore, exploring treatments for negative and cognitive symptoms of TRS will also be a future research direction in the field of TRS.

#### Early-onset schizophrenia and early identification of TRS

TRS is a serious mental disorder and one of the main causes of dysfunction and disability in patients; however, the treatment method for TRS is a tricky issue in current clinical practice. Clozapine is the only effective evidence-based drug available for the treatment of TRS. Guidance from the National Institute for health and Care Excellence (NICE) recommend that people with schizophrenia be offered clozapine if their psychotic symptoms do not significantly improve despite the use of ≥2 different antipsychotics in adequate doses (NICE, 2014). However, it is currently impossible for clinicians to accurately predict which patients will not respond to first-line antipsychotic treatment prior to medication administration, making early identification of TRS particularly important given that this has the potential to improve clinical outcomes as early as possible and to reduce social and functional disability due to TRS (Wheeler et al., 2009; Stroup et al., 2016). Lally et al. (2016) followed 246 patients with first-episode schizophrenia for 5 years to assess the clinical and demographic risk factors associated with the emergence of TRS. The results showed that 34% of the patients eventually developed refractory disease, with 70% of patients failing to respond to antipsychotic medication from onset. Furthermore, they found that patients with an earlier age of onset (<20 years) were more likely to develop refractory disease later in life than those with nonrefractory onset. Other studies support the idea that early-onset schizophrenia (EOS) has a worse prognosis than adult-onset schizophrenia (Röpcke and Eggers, 2005; Vyas et al., 2007).

EOS is defined as schizophrenia with an age of onset of 13–17 years (Clemmensen et al., 2012), and a lower prevalence rate than in the general population (Jerrell and McIntyre, 2016; Driver et al., 2020). However, negative symptoms are more common (Rammou et al., 2019), symptoms are more severe (Coulon et al., 2020), and prognosis is poorer in patients with EOS compared to patients with schizophrenia presenting in adulthood (Díaz-Caneja et al., 2015; Stentebjerg-Olesen et al., 2016; Immonen et al., 2017). More importantly, the risk of developing drug resistance is higher (Smart et al., 2021; Iasevoli et al., 2022; Siskind et al., 2022). Nevertheless, this particular group of adolescents is in a period of growth and development, and early manifestations of schizophrenia, neurodevelopmental comorbidities, and other psychiatric disorders may be difficult to differentiate, leading to misdiagnosis and underdiagnosis being more common. Therefore, timely and accurate diagnosis of schizophrenia in adolescents is of great significance for the detection of TRS.

Antipsychotics are currently the first-line clinical treatment for EOS in adolescents (Abidi et al., 2017). An umbrella review from Correll et al. reviewed the safety and tolerability of a variety of medications in children/adolescents with psychiatric disorders and found that antipsychotics (first- or second-generation antipsychotics) were effective for EOS (Correll et al., 2021). Although medication is safer for patients with EOS (Solmi et al., 2020), adolescents are more sensitive to medication side effects compared with adults (Liu et al., 2019). Currently available medications approved for use in adolescents with schizophrenia are limited (Lopez-Morinigo et al., 2022; Smogur et al., 2022), and extra-approved medications will be used in clinical practice after informing the guardian and obtaining his or her consent. Second-generation antipsychotics, such as olanzapine and risperidone, are currently preferred in the clinic; however, their effects on metabolic and endocrine functions should be carefully considered before use. Recent studies suggest that clozapine is more effective than other antipsychotics in the treatment of EOS (Arango et al., 2020; Correll et al., 2021; Adnan et al., 2022), even for refractory EOS (Schneider et al., 2014), with clozapine exhibiting the best results. Age-related differences in drug metabolism and adverse effects must also be considered. In addition, for patients who do not respond to early treatment with antipsychotics, a change in medication or an appropriate dose increase should be considered. However, the identification of TRS and early use of clozapine in patients with EOS remains a concern. All current randomised controlled trials of antipsychotics have been conducted in adult patients, and there is a lack of evidence in patients with EOS.

Based on our findings, the hotspots for future research will include methods for accurately diagnosing EOS at an early stage, identification of patients who may develop refractory TRS at a later stage, selection of reasonable and appropriate antipsychotics, and how to target specific age groups and assign individualised treatment regimens.

### Limitations

This study had some limitations. First, clearly many articles that had been accepted in 2024 but not yet published were not be included in the analysis; therefore, it is possible that we underestimated their impact. And bibliometrics usually focuses on formally published literature and ignores the contribution of informal publications such as book, conference papers, preprints, etc. Second, owing to the limitations of the functionality of the software, we only searched for literature in English, potentially missing the contributions of non-English-speaking countries. Third, bibliometrics may be underdeveloped for emerging and cross-cutting disciplines related to TRS, as research in these areas may not yet have developed a stable citation network and recognized evaluation criteria, so we may have overlooked their importance.

## Conclusion

The number of publications in treatment-resistant schizophrenia (TRS) has been on the rise in recent years, indicating that the field is attracting more and more researchers. The United States ranks first in the number of publications and has more collaborations with other countries. Schizophrenia Research and American Journal of Psychiatry are the journals with the most published articles and the most co-citations, respectively, and future research is likely to focus on areas such as PSYCHOLOGY/EDUCATION areas. However, issues such as how to use antipsychotics more effectively to treat TRS and how to predict the emergence of TRS as early as possible are still in urgent need of investigation and are current challenges for clinicians. The results of this study predicted and analyzed the future research hotspots and showed that cognitive impairment, clozapine resistant schizophrenia, early-onset schizophrenia and early recognition of TRS may attract more researchers’ attention in the future. Also, this study helps researchers to identify appropriate research directions and partners.

## Data Availability

The raw data supporting the conclusions of this article will be made available by the authors, without undue reservation.
